# TIPRL potentiates survival of lung cancer by inducing autophagy through the eIF2α-ATF4 pathway

**DOI:** 10.1038/s41419-019-2190-0

**Published:** 2019-12-20

**Authors:** Su-Jin Jeon, Jun-Ho Ahn, Debasish Halder, Hyun-Soo Cho, Jung-Hwa Lim, Soo Young Jun, Jeong-Ju Lee, Ji-Yong Yoon, Min-Hyuk Choi, Cho-Rok Jung, Jin-Man Kim, Nam-Soon Kim

**Affiliations:** 1 Genome Research Center, Korea Research Institute of Bioscience and Biotechnology (KRIBB), 125 Gwahak-ro, Yuseong-gu, Daejeon, Republic of Korea; 2 Department of Functional Genomics, KRIBB School of Bioscience, University of Science and Technology (UST), 217 Gajeong-ro, Yuseong-gu, Daejeon, Republic of Korea; 3 Department of Predictive Toxicology, Korea Institute of Toxicology, 141 Gajeong-ro, Yuseong-gu, Daejeon, Republic of Korea; 4 Gene Therapy Research Unit, Korea Research Institute of Bioscience and Biotechnology, 125 Gwahak-ro, Yuseong-gu, Daejeon, Republic of Korea; 5 Department of Pathology, School of Medicine, Chungnam National University, 99 Daehak-ro, Yuseong-gu, Daejeon, Republic of Korea

**Keywords:** Non-small-cell lung cancer, Stress signalling

## Abstract

Autophagy, an intracellular system of degrading damaged organelles and misfolded proteins, is essential for cancer cell survival. Despite the progress made towards understanding the mechanism, identification of novel autophagy regulators presents a major obstacle in developing anticancer therapies. Here, we examine the association between the TOR signaling pathway regulator-like (TIPRL) protein and autophagy in malignant transformation of tumors. We show that TIPRL upregulation in non-small cell lung cancer (NSCLC) potentiated autophagy activity and enabled autophagic clearance of metabolic and cellular stress, conferring a survival advantage to cancer cells. Importantly, the interaction of TIPRL with eukaryotic initiation factor 2α (eIF2α) led to eIF2α phosphorylation and activation of the eIF2α-ATF4 pathway, thereby inducing autophagy. Conversely, TIPRL depletion increased apoptosis by reducing autophagic clearance, which was markedly enhanced in TIPRL-depleted A549 xenografts treated with 2-deoxy-D-glucose. Overall, the study indicated that TIPRL is a potential regulator of autophagy and an important drug target for lung cancer therapy.

## Introduction

Autophagy promotes cancer growth and progression by alleviating metabolic stress and by enhancing the supply of nutrients via the degradation of cellular organelles and unfolded proteins^1^. Several studies have demonstrated that autophagy can act as both a suppressor and an activator of cancer. Rao et al. demonstrated that autophagy suppression promoted tumor initiation in murine lung cancer; however, continued suppression eventually reduced tumor masses, suggesting the essential and sensitive role of autophagy in cancer progression^2^. This multifaceted role makes autophagy an attractive target in cancer research. Therefore, it would be advantageous to explore the underlying mechanisms linking autophagy to cancer to develop effective cancer therapies.

Autophagy is an important adaptive mechanism for cancer cells, because cancer cells demand higher metabolic and biosynthetic activities. Mitophagy has been reported to promote cancer cell survival by relieving cellular stress resulting from dysfunctional mitochondria ascribed to excess accumulation of reactive oxygen species (ROS) and reactive nitrogen species (RNS)^3,4^. In addition, aggrephagy relieves endoplasmic reticulum (ER) stress that occurs from the build-up of unfolded proteins in the ER through the unfolded-protein response (UPR) pathway^5,6^. The failure of autophagy to mitigate mitochondria-originated and ER-originated stresses results in cancer cell death^7–9^. Cancer cells also bypass the cellular checkpoints, thereby preventing cell cycle arrest and subsequent apoptosis to increase their proliferation and aggressiveness^10,11^. Therefore, understanding the mechanisms underlying autophagy is important for mitigating stresses in cancer cells.

Mammalian target of rapamycin (mTOR) plays a crucial role in sensing metabolic stress and regulates important physiological functions, including cell growth, proliferation, and autophagy. Under conditions of nutrient stress, autophagy functions via the mTOR pathway to promote metabolic homeostasis and survival of cancer cells^12^. Previously, our group demonstrated that the TOR signaling pathway regulator-like (TIPRL) protein was upregulated in liver cancers, which resulted in the TRAIL resistance of cancer cells via de-phosphorylation of MKK7^13^. Several studies have demonstrated the relationship between TIPRL and mTOR in cancer. In yeast cells, TIPRL is involved in mTOR-dependent autophagy^14^. Another study showed that TIPRL potentiated mTORC1 signaling in human cancer cell lines, depending on amino acid availability^15^. Based on these observations, we studied the mechanism underlying the involvement of TIPRL in autophagy.

Autophagy induction causes cancer cells to develop resistance to anticancer drugs. It was previously reported that eIF2α is phosphorylated in response to bortezomib-induced ER stress, and thus initiates autophagy by activating the ATF4 (which controls transcription of autophagy-related genes)^16,17^. These studies highlight the critical role of the eIF2α-ATF4 pathway in controlling autophagy induction upon stress conditions.

In this study, we discover a novel molecular process in which TIPRL activates autophagy thereby inhibiting metabolic stress-induced apoptosis in lung cancer cells. TIPRL acts as a major player in stress-induced autophagy by directly interacting with eIF2α and inducing eIF2α phosphorylation, while TIPRL knockdown makes cancer cells more susceptible to environmental stress. Overall, our results strongly indicate that targeting TIPRL could be a potential route for the development of anti-lung cancer therapies.

## Results

### TIPRL expression levels are correlated with malignancy in non-small cell lung cancer

**Fig. 1:**
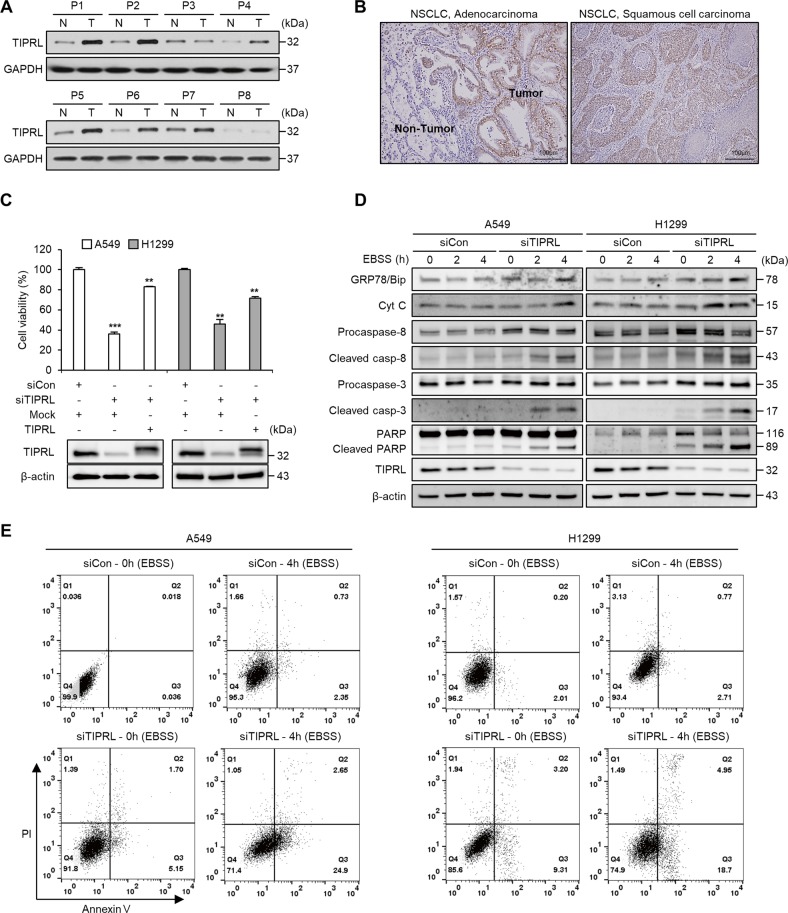
Depletion of TIPRL upregulated in cancer tissues induces apoptosis under metabolic stress. **a** Detection of TIPRL in paired samples of lung adenocarcinoma (T) and normal lung tissues (N). Stage of tumor tissues from patients: P1 (Ia), P2 (Ia), P3 (Ib), P4 (Ib), P5 (IIa), P6 (IIa), P7 (IIb), and P8 (IIb). **b** Immunohistochemistry assay of NSCLCs was performed using anti-TIPRL antibody. **c** MTT assay of A549 and H1299 cells at 48 h after transfection with siCon and siTIPRL or cotransfection with siTIPRL/HA-TIPRL plasmid. **d** Western blots of apoptosis pathway proteins and ER stress marker GRP78 of A549 and H1299 cells after incubated in EBSS for 2 or 4 h after transfection with siCon or siTIPRL. **e** FACS analysis of Annexin V and PI stained A549 and H1299 cells incubated in EBSS for 4 h following transfection with siCon or siTIPRL. Depicted western blots are representatives from 2–3 independent experiment. All quantitative bar data are mean ± SEM. *p*-value was calculated by *t*-test. **P < 0.01, ***P < 0.001.

First, we examined the levels of TIPRL in clinical lung cancer specimens, and found that tumor tissues had significantly higher levels of TIPRL than normal tissues (Fig. 1a). Immunohistochemistry (IHC) analysis of 179 paired lung cancer and normal tissues showed that NSCLCs exhibited positive staining for TIPRL (Fig. 1b). In addition, we found a strong positive association between TIPRL levels and the tumor stages (Supplementary Table. S1). Next, we performed an expression analysis of small-interfering RNA (siRNA)-mediated TIPRL-knockdown in A549 cells. Unexpectedly, an analysis using the GO database and PANTHER classification revealed changes in the cellular process (28.9%) and metabolic process (21.9%) of TIPRL-knockdown cells (Supplementary Fig. S1a). In addition, the KEGG pathway analysis identified that TIPRL knockdown is highly associated with the metabolic pathway and p53 signaling pathway (Supplementary Fig. S1b). These results demonstrate the association between TIPRL and cancer malignancy, and suggest that an overexpression of TIPRL could potentially confer metabolic benefits to lung cancer cells, helping malignant cells to survive.

### TIPRL knockdown induces cell death by activating apoptosis in lung cancer cells under metabolic stress

To gain insight into the role of TIPRL in lung cancer progression, we examined the impact of TIPRL knockdown on the proliferation in A549 and H1299 cells through MTT assays. We found that knockdown of TIPRL led to the inhibition of cancer cell growth; this effect was partially reversed when co-transfected with HA-tagged TIPRL plasmid (Fig. 1c). Since TIPRL is reported to be closely related to the mTOR pathway, we employed Earle’s balanced salt solution (EBSS) as a starvation model to induce metabolic stress and elucidate the regulation mechanism of TIPRL on cancer cell growth. An analysis of the apoptosis signaling pathway shows that levels of GRP78/BiP and cytochrome C release are significantly higher in the TIPRL-knockdown cells following EBSS incubation. Moreover, TIPRL-knockdown cells have higher levels of cleaved caspase-8, caspase-3, and poly ADP ribose polymerase (PARP; Fig. 1d). In congruence, FACS analysis with annexin V and PI staining revealed the following results after TIPRL knockdown and 4 h of EBSS incubation: the percentages of A549 cells exhibiting early apoptosis changed from 0.036% to 24.9%, while late apoptosis changed from 0.018% to 2.65%. In H1299 cells, the percentages of cells exhibiting early apoptosis changed from 2.01% to 18.7%, while late apoptosis changed from 0.2% to 4.95% (Fig. 1e). These results indicate that under metabolic stress, TIPRL acted as a crucial factor for cancer cell survival. Therefore, TIPRL knockdown caused lung cancer cells to become more susceptible, promoting cell death by activation of apoptotic signaling.

### TIPRL knockdown inhibits autophagy by preventing autophagosome formation in lung cancer cells under metabolic stress

**Fig. 2:**
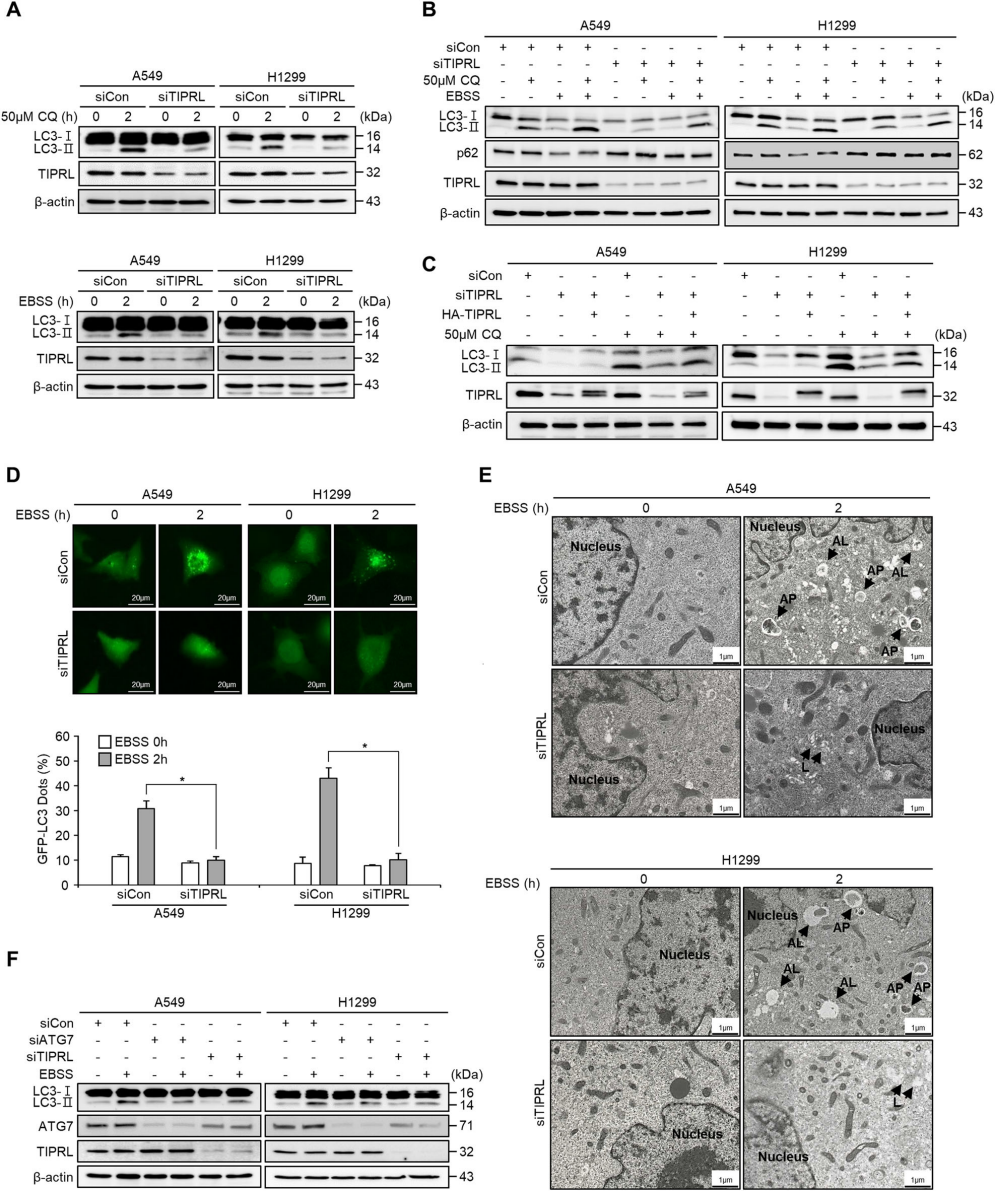
TIPRL knockdown inhibits autophagy through prevention of autophagosome formation under metabolic stress. **a** Detection of LC3II by western blots of A549 and H1299 cells treated with 50 µM chloroquine for 2 h (upper panel) or with Earl’s Balanced Salt Solution (EBSS) for 2 h (lower panel) after transfection with siCon or siTIPRL. **b** Western blots of LC3II and p62 protein of A549 and H1299 cells after incubating in 50 µM chloroquine and/or EBSS for 2 h after transfection with siCon or siTIPRL. **c** Detection of LC3II by western blots of A549 and H1299 cells treated with 50 µM chloroquine after transfection of siCon and siTIPRL or cotransfection with siTIPRL/HA-TIPRL plasmid DNA. **d** GFP-LC3 puncta assays of A549 and H299 cells incubated in EBSS for 2 h after transfection with EGFP-LC3 plasmid and siCon or siTIPRL. The percentages of cells showing autophagy were quantified by counting the number of cells positive for GFP-LC3 punctae in 200 GFP-positive cells to have enough sample size and to calculate the standard deviation. **e** TEM images of A549 and H1299 cells transfected with siCon or siTIPRL and then incubated in EBSS. Autophagosomes are labeled as ‘AP’, autophagolysosomes are indicated as ‘AL’ and lysosomes are labeled as ‘L’. Scale bar = 1 µm. **f** Western blots of LC3II protein of A549 and H1299 cells after incubation in EBSS for 2 h following transfection with siTIPRL or siATG7. Depicted western blots are representatives from 2–3 independent experiment. All quantitative bar data are mean ± SEM. p-value was calculated by *t*-test. *P < 0.05.

Based on our results, we examined the effects of TIPRL knockdown on autophagy levels in order to investigate their relationship in lung cancer. Interestingly, we found that LC3-II levels were significantly lowered in TIPRL-knockdown A549 and H1299 cells after treatment with either chloroquine or EBSS (Fig. 2a), as well as when subjected with both treatments (Fig. 2b). This indicates that TIPRL depletion inhibited basal level and EBSS-induced autophagy by preventing the accumulation of autophagosomes. However, the TIPRL-mediated autophagy inhibition was successfully recovered by an exogenous transfection of TIPRL (Fig. 2c). This data suggests that TIPRL regulates autophagy at a very early stage, possibly during the stage of autophagosome formation.

The inhibition of autophagy by TIPRL was further confirmed by measuring the numbers of GFP-LC3 puncta, which were remarkably reduced by TIPRL knockdown in both A549 (from 30.8% to 11.5%) and H1299 cells (from 43% to 10.25%) treated with EBSS (Fig. 2d). Transmission electron microscopy (TEM) analysis of TIPRL-knockdown cells under starvation showed that the number of autophagosomes and autolysosomes were significantly decreased (Fig. 2e). These results indicate that TIPRL may be associated with signaling processes required for autophagosome formation.

In addition, we compared the knockdown effects of ATG7 and TIPRL under metabolic stress, as ATG7 plays a pivotal role in homeostasis, and lowering ATG7 expression can inhibit autophagy^18^. Figure 2f clearly indicates that TIPRL knockdown significantly inhibited autophagy like that observed in ATG7 knockdown. Interestingly, we observed that protein levels of ATG7 were reduced by TIPRL knockdown. These findings suggest that TIPRL functioned as a key regulator of autophagy by modulating autophagosome formation via ATG7 levels in lung cancer cells.

### TIPRL knockdown inhibits autophagic clearance and ATP recycling, thus inducing cell cycle arrest

**Fig. 3:**
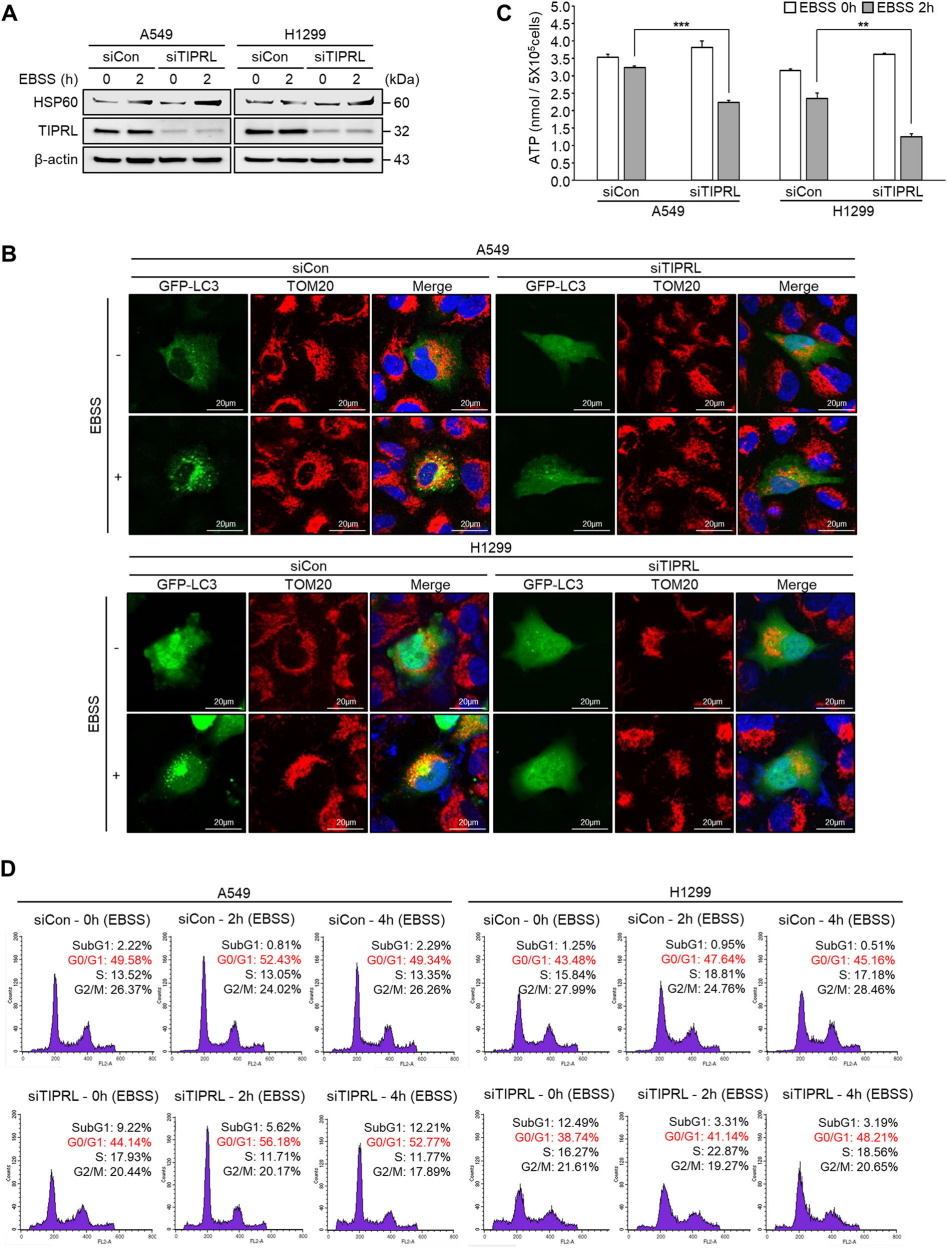
TIPRL knockdown increases susceptibility to cellular stress, and inhibits autophagic clearance under metabolic stress. **a** Detection of ER stress marker HSP60 by western blots of A549 and H1299 cells after transfection with siCon or siTIPRL and then incubated in EBSS. **b** Double immunostaining of GFP-LC3 and mitochondria using anti-TOM20 antibody in A549 and H1299 cells incubated in EBSS for 2 h after transfection with siCon or siTIPRL; yellow indicates colocalization of autophagosome and mitochondria. **c** Detection of cellular ATP in A549 and H1299 cells incubated in EBSS for 2 h following transfection with siCon or siTIPRL. We repeated this three times to calculate the standard deviation. **d** PI staining of A549 and H1299 cells treated with EBSS for 2 h and 4 h following transfection with siCon or siTIPRL, the portion of cells in each phase is stated. Depicted western blots are representatives from 2–3 independent experiment. All quantitative bar data are mean ± SEM. *p*-value was calculated by *t*-test. **P < 0.01, ***P < 0.001.

To evaluate whether TIPRL-mediated autophagy has a role in the clearance mechanisms of cells, we examined the levels of the ER stress marker HSP60. We found an increase of HSP60 expression in TIPRL-knockdown cells, indicating an accumulation of unfolded proteins upon EBSS treatment (Fig. 3a). In addition, we observed that the colocalization of GFP-LC3 puncta and TOM20 (mitochondrial marker) was clearly apparent in control cells incubated with EBSS, indicating mitophagy activity under starvation. However, the colocalization was markedly reduced in both cell lines by TIPRL knockdown (Fig. 3b). Moreover, we studied the effects of TIPRL knockdown on aggrephagy by measuring TX-100-insoluble p62, which represents ubiquitinated-p62^19^. Treatment with chloroquine or EBSS in control cells exhibited increased TX-100-insoluble p62, while TIPRL knockdown cells demonstrated even higher levels of TX-100-insoluble p62, indicating significant accumulation of p62-tagged unfolded proteins (Supplementary Fig. S2a). Overall, these findings suggest that TIPRL-deficient cells disturbed the autophagy activity and failed to eliminate critical cellular stress.

The level of cellular ATP is an important parameter for monitoring de novo energy recycling in the autophagic process^20,21^. We observed that autophagy inhibition by TIPRL knockdown led to a drastic decrease in ATP recycling during starvation (Fig. 3c). Moreover, a substantial G0/G1 cell cycle arrest was observed in TIPRL-knockdown lung cancer cells treated with EBSS. In control cells, even though the portion of cells in G0/G1 arrest was slightly increased after 2 h treatment with EBSS (49.58% to 52.43% and 43.48% to 47.64%, respectively), after 4 h the proportions of G0/G1 arrest cells were successfully recovered. However, in TIPRL-knockdown A549 cells, the proportion of cells in G0/G1 cell cycle arrest was significantly increased after 2 h (from 44.14% to 56.18%) and was not fully recovered after 4 h. Notably, in TIPRL-knockdown H1299 cells, treatment with EBSS for 2 h slightly increased the proportion of cells in G0/G1 arrest (from 38.74% to 41.14%), but prolonged starvation further increased the proportion of cells in G0/G1 arrest (up to 48.21%; Fig. 3d). This data indicates that TIPRL can oversee the stress-adaptation processes of cancer cells by regulating autophagy activity.

### TIPRL induces autophagy through the eIF2α-ATF4 pathway under metabolic stress

**Fig. 4:**
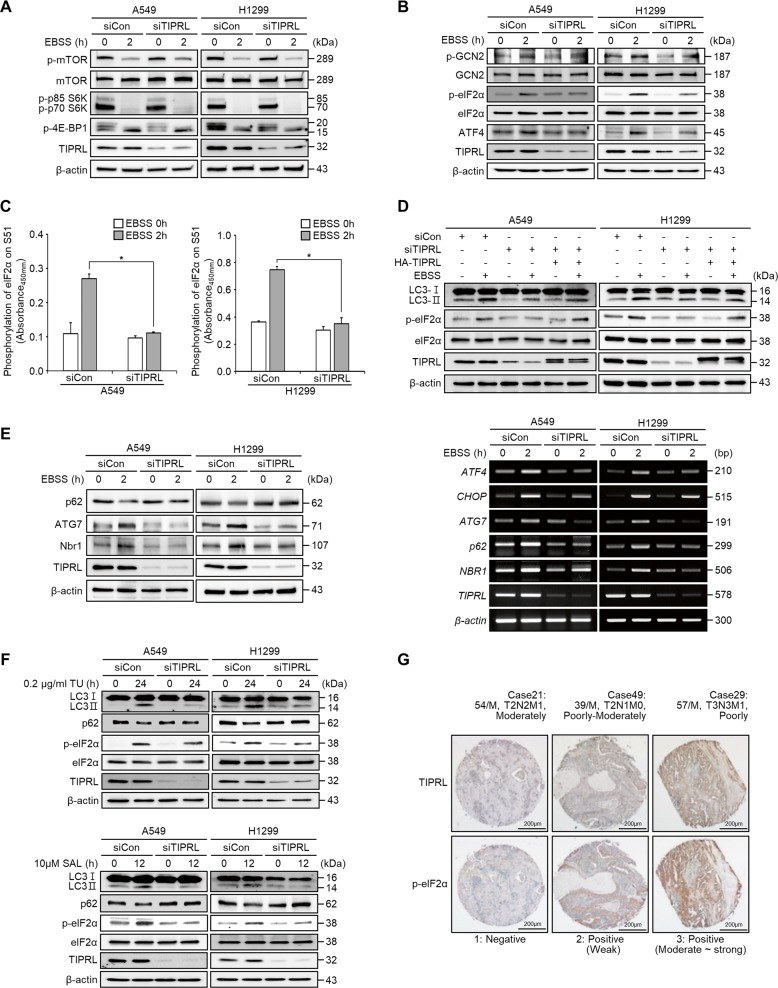
TIPRL induces autophagy through the eIF2α-ATF4 pathway under metabolic stress. **a**, **b** Detection of mTOR pathway proteins (**a**) and autophagy pathway proteins (**b**) by western blots of A549 and H1299 cells after incubation in EBSS for 2 h following transfection with siCon or siTIPRL. **c** Detection of phosphorylated-eIF2α by ELISA assay. We repeated this assay three times to calculate the standard deviation. **d** Western blots of LC3II and phospho-eIF2α proteins of A549 and H1299 cells incubated in EBSS for 2 h after transfection with siCont and siTIPRL or co-transfection with siTIPRL/HA-TIPRL plasmid DNA. **e** Detection of autophagy-related genes by western blots (left panel), or RT-PCR (right panel) in A549 and H1299 cells incubated in EBSS for 2 h after transfection with siCon or siTIPRL. **f** Western blots of LC3II, p62, and phospho-eIF2α proteins of A549 and H1299 cells treated with tunicamycin (0.2 µg/ml) for 24 h or salubrinal (10 µM) for 12 h after siCon or siTIPRL transfection. **g** Immunohistochemical analysis of TIPRL and phospho-eIF2α protein levels in human lung cancer tissues. Depicted western blots are representatives from 2–3 independent experiment. All quantitative bar data are mean ± SEM. *p*-value was calculated by *t*-test. * P < 0.05.

Considering the mTOR is one of the main pathways regulating autophagy and TIPRL is known to positively regulate the mTORC1 pathway^22,23^, we assessed changes in the mTOR pathway following TIPRL knockdown. To this end, we examined the phosphorylation levels of mTOR, ribosomal protein S6 kinase 1 (S6K1), and translation initiation factor 4E-binding protein 1 (4E-BP1) and found no differences in mTOR signaling regulators between control and TIPRL-knockdown cells (Fig. 4a). These results indicate that autophagy downregulation in TIPRL-deficient cells was independent of mTOR signaling, contrary to previous reports^14,22^.

Because ER stress and nutritional stress regulate autophagy through activation of eIF2α phosphorylation at Ser51^24–26^, we monitored the levels of eIF2α pathway-related proteins. While control cells showed increased levels of phosphorylated eIF2α and GCN2, as well as ATF4, TIPRL-knockdown cells showed significantly lowered expression of these regulator proteins under starvation (Fig. 4b). This result was further confirmed through quantitative p-eIF2α (Ser51) ELISA analysis (Fig. 4c), and transfection of cells with HA-tagged TIPRL reversed the inhibition of eIF2α phosphorylation caused by TIPRL knockdown (Fig. 4d). These results indicate that TIPRL is necessary for autophagy induction by upregulating eIF2α phosphorylation.

In addition, we found that EBSS incubation increased the transcription levels of eIF2α-ATF4 pathway-regulated genes such as *ATF4, CHOP, ATG7, p62*, and *NBR1*, while TIPRL knockdown mitigated transcriptions of autophagy-related genes (Fig. 4e; right panel). Consistent with the transcription levels, the protein levels of ATG7 and NBR1 were decreased in TIPRL-knockdown cells upon starvation. However, p62 in control cells was degraded upon EBSS treatment, while in TIPRL-knockdown cells, it was not degraded due to autophagy inhibition (Fig. 4e; left panel). This finding was supported by a time-course EBSS treatment experiment, where *p62* transcription was increased as starvation continued, but p62 protein was degraded upon autophagy induction (Supplementary Fig. S3a). These findings indicate that decreased eIF2α phosphorylation is due to the loss of TIPRL function, which ultimately inhibits the expression of essential autophagy-related genes.

Metabolic stress caused by hypoxia has been shown to induce autophagy via the PERK-dependent pathway^27^. To determine the role of TIPRL in hypoxia-induced autophagy, we incubated H1299 cells in 1% O_2_. We found that TIPRL-knockdown cells mitigated the autophagy process through inhibition of the eIF2α-ATF4 pathway under hypoxia (Supplementary Fig. S3b). Similar observations were also made for TIPRL-knockdown cells treated with tunicamycin and salubrinal, which are known, respectively, as an ER stress inducer and a p-eIF2α specific inhibitor. eIF2α phosphorylation and the eIF2α-ATF4 pathway-regulated genes such as ATG7, p62, and NBR1 were attenuated in TIPRL-knockdown cells treated with tunicamycin or salubrinal (Fig. 4f, Supplementary Fig. S3c).

In addition, we examined the relationship between TIPRL expression and eIF2α phosphorylation patterns in 41 microarray samples of human lung cancer tissues, using immunohistochemistry. Our results showed that TIPRL expression and eIF2α phosphorylation were positively correlated (*r* = 0.53956, *P* < 0.001215) (Fig. 4g). Altogether, the data suggests that TIPRL is essential for autophagy induction through the eIF2α-ATF4 pathway upon various stress conditions that evoke autophagy machinery.

### The direct interaction between TIPRL and eIF2α increase phosphorylation of eIF2α

**Fig. 5:**
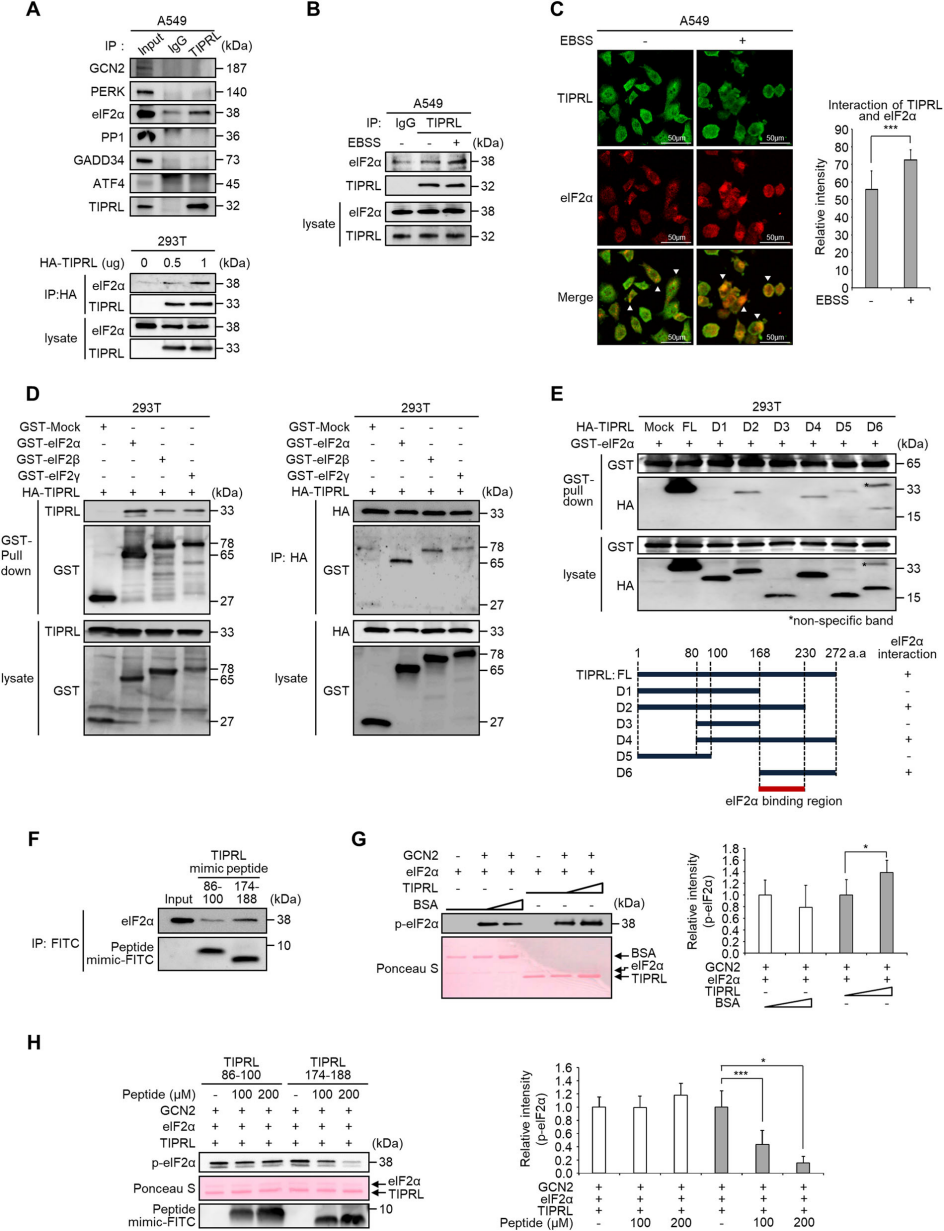
Interaction between TIPRL and eIF2α increases phosphorylation of eIF2α. **a** IP assays of cell lysates of A549 cells with anti-TIPRL antibody (upper panel) and cell lysates of 293 T cells with anti-HA antibody after transfection with gradual concentration of HA-tagged TIPRL (lower panel). **b** IP assays with anti-TIPRL antibody in A549 cells incubated in EBSS for 2 h. **c** Immunocytochemistry data of A549 cells where TIPRL and eIF2α proteins were double-immunostained and treated with goat anti-rabbit mouse IgG-FITC (green) and bovine anti-mouse IgG-Texas Red (red). Relative intensity of interaction were measured. **d** GST-pull down assay (left panel) and IP assay (right panel) with anti-HA antibody of 293 T cells co-transfected with HA-TIPRL along with GST-Mock, GST-eIF2α, GST-eIF2β, and GST-eIF2γ, respectively. **e** GST-pull down assay of 293T cells co-transfected with GST-eIF2α and TIPRL deletion mutants (D1–D6) (upper panel); six deletion fragments of TIPRL were sub-cloned into the pCGN-HA vector (lower panel). **f** IP assay of A549 cells mixed with TIPRL-mimic peptides which represent the regions of TIPRL spanning the amino acids 86–100 or 174–188. **g** In vitro kinase assays of phospho-eIF2α using recombinant eIF2α, gradual concentration of TIPRL, and GCN2 proteins. Phosphorylation level of eIF2α was detected by Western blots, and recombinant proteins were stained using Ponceau S (left panel). Relative intensity of phospho-eIF2α were measured (right panel). **h** In vitro kinase assays of phospho-eIF2α using recombinant eIF2α, GCN2, and TIPRL proteins with incubation of 100 and 200 µM of TIPRL-mimic peptides. Phosphorylation of eIF2α and transfection of peptide were detected by Western blots, and recombinant proteins were stained using Ponceau S (left panel). Relative intensity of phospho-eIF2α were measured (right panel). Depicted western blots are representatives from 2–3 independent experiment. All quantitative bar data are mean ± SEM. *p*-value was calculated by *t*-test. *P < 0.05, ***P < 0.001.

To identify proteins that may interact with TIPRL to regulate eIF2α phosphorylation, we performed immunoprecipitation (IP) assays. IP analysis revealed that only eIF2α clearly interacted with TIPRL in A549 cells among several candidate proteins (Fig. 5a, upper panel). These findings were further strengthened by demonstrating a strong interaction between overexpressed HA-tagged TIPRL and endogenous eIF2α in 293T cells in a concentration-dependent manner (Fig. 5a, lower panel). Furthermore, endogenous binding of TIPRL and eIF2α in A549 cells increased when cells were incubated in EBSS (Fig. 5b). In addition, through an immunocytochemical analysis, we observed that colocalization of TIPRL and eIF2α was significantly increased when treated with EBSS (Fig. 5c). Considering that eIF2α is a member of the eIF2 complex comprised of three component (including eIF2β and eIF2γ), we performed GST-pull down and IP assays to specify the exact binding subunit among the eIF2 complex and found that eIF2α dominantly interacted with TIPRL (Fig. 5d).

To identify the TIPRL regions involved in binding with eIF2α, we used six HA-tagged truncated mutant plasmids of TIPRL^13^. Plasmids encoding GST-eIF2α and truncated TIPRL mutants were co-transfected into 293T cells to perform a GST-pull down assay. The results revealed that eIF2α interacted with the D2, D4, and D6 mutants of TIPRL, indicating that the region including amino acids 168–230 of the TIPRL protein was involved in eIF2α binding (Fig. 5e). Furthermore, a fluorescein isothiocyanate (FITC)-tagged synthetic peptide toward the eIF2α-binding region of TIPRL (174–188 amino acids) clearly interacted with eIF2α, whereas a negative control peptide (86–100 amino acids) did not (Fig. 5f). In addition, an in vitro kinase assay revealed that the levels of p-eIF2α were increased in the presence of TIPRL in a dose-dependent manner (Fig. 5g). Unexpectedly, treatment with the TIPRL mimic peptide resulted in significantly inhibited eIF2α phosphorylation, indicating this peptide inhibited TIPRL binding to eIF2α and is possibly capable of autophagy inhibition (Fig. 5h). However, mimic peptide in the absence of TIPRL had no effects on eIF2α phosphorylation (Supplementary Fig. S4a). These results indicate that the interaction between TIPRL and eIF2α was responsible for the increase in eIF2α phosphorylation, and TIPRL mimic peptide has a potential of autophagy inhibition.

### TIPRL knockdown augments the growth-inhibitory effects of 2-deoxy-D-glucose (2-DG) in lung cancer xenografts

**Fig. 6:**
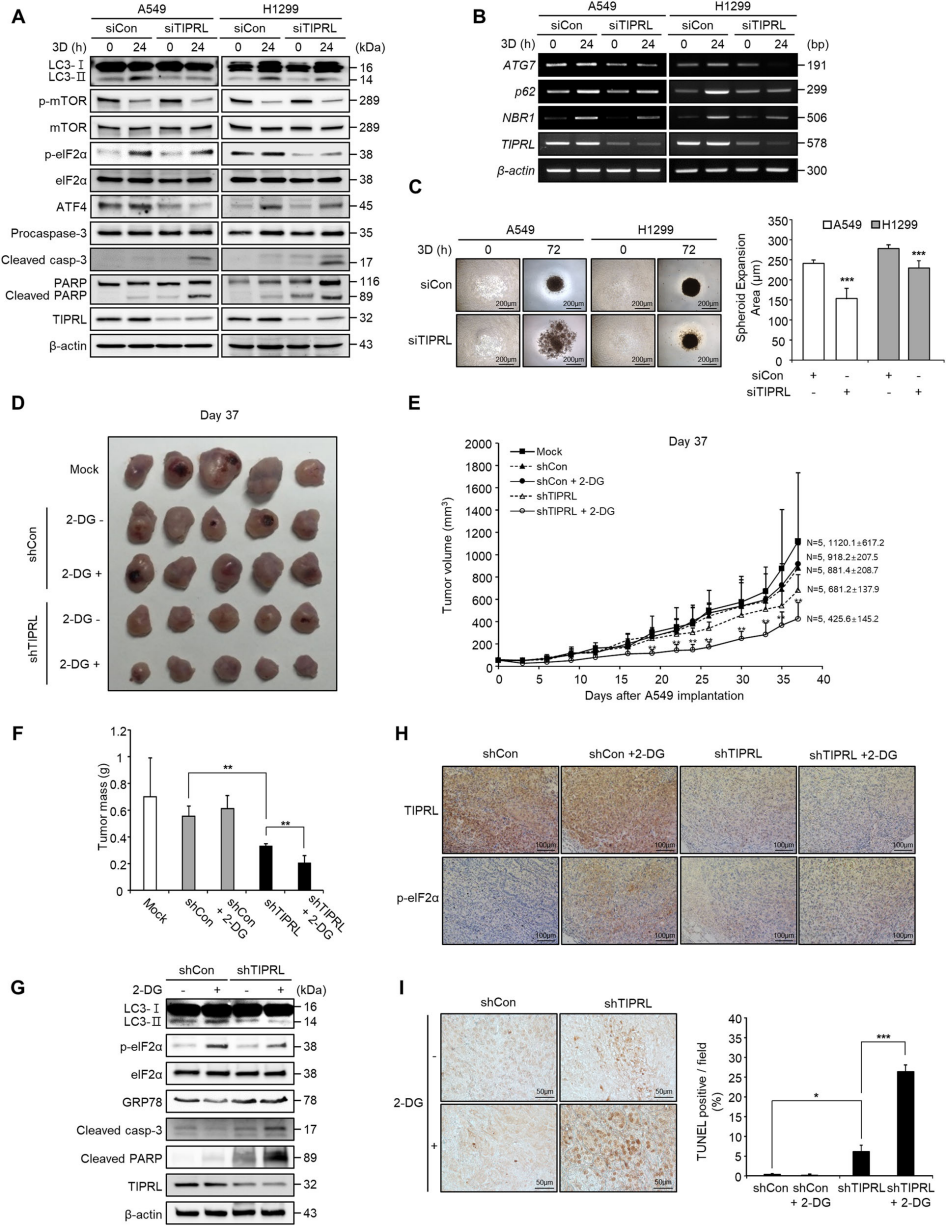
TIPRL knockdown enhances the growth inhibitory effects of 2-deoxy-D-glucose (2-DG) on A549 tumor xenografts. Western blots of autophagy and apoptosis pathway proteins (**a**) and RT-PCR of autophagy-related genes (**b**) in A549 and H1299 cells cultured by hanging drop method (3D culture) for 24 h. **c** Microscopy images of A549 and H1299 cells cultured in Ultra Low Attachment plates. The spheroid expansion area was quantified by measuring the size of spheroids. **d–i** A549 cells (5 × 10^6^ cells) were injected into nude mice (*n* = 5 per group) subcutaneously to induce the growth of A549 tumor xenografts. After 2 weeks, the mice were intraperitoneally injected with either 2-DG (1000 mg/kg, in 100 µl of PBS/mouse) (+2-DG) or PBS (−2-DG); following this, the xenografts were injected intratumorally with the lentivirus scramble shRNA or lentivirus-mediated TIPRL shRNA (1.5 × 10^8^ PFU (TD)/mouse). Tumor-bearing mice were photographed at 37 days post-injection (**d**). The tumor volume was measured every 3 days (**e**). At the end of the experiment, tumors were excised from the mice and weighed (**f**). Detection of autophagy and apoptosis pathway proteins by western blots from lysates of each group of mice (**g**). Paraffin-embedded sections from tumors of each group were subjected to immunohistochemistry (**h**) and TUNEL assays (**i**). The percentages of TUNEL positive cells were quantified by counting cells positive for staining to calculate the standard deviation. Depicted western blots are representatives from 2–3 independent experiment. All quantitative bar data are mean ± SEM. *p*-value was calculated by *t*-test. *P < 0.05, **P < 0.01, ***P < 0.001.

3D culture systems can imitate the in vivo tumor-formation process and provide a hostile microenvironment^28^. Thus, we used a 3D culture system as an ex vivo model to confirm the possibility of autophagy regulation through TIPRL. After 24 h of hanging-drop culture, we observed that TIPRL knockdown mitigated the autophagy process through the eIF2α-ATF4 pathway and increased apoptosis incidence (Fig. 6a, b). In addition, TIPRL-knockdown cells cultured in Ultra Low Attachment (ULA) plates formed looser and significantly smaller spheroid mass (Fig. 6c). These results demonstrate that TIPRL plays a crucial role in the formation of spheroids and suggest a possibility for targeting TIPRL in an in vivo environment.

2-deoxy-D-glucose (2-DG), an inhibitor of glycolysis, has been reported to induce starvation and autophagy in cancer cells^29^. In A549 cells, we observed that TIPRL knockdown significantly augmented the growth-inhibitory effects of 2-DG (Supplementary Fig. S5a). To confirm our results in an in vivo model, we constructed lentiviral vectors encoding short hairpin RNA (shRNA) against TIPRL (shTIPRL) and control shRNA (shCon). As shown in Fig. 6d, e, shTIPRL treated mice exhibited significantly lower tumor growth than that of shCon treated mice, and the tumor growth-suppressive effects of 2-DG were more pronounced in the shTIPRL group. A slight reduction of tumor growth in the control group when compared with the mock group (PBS treatment) is considered to be a side effect of lentiviral treatment. In addition, transfection with shTIPRL significantly lowered tumor masses by 70.7% and by 52.5% with and without 2-DG treatment, respectively. However, transfection with shCon did not significantly alter the effects of 2-DG on tumor growth (Fig. 6f). No significant differences in body weight were observed (Supplementary Fig. S5b).

Western blots of A549 xenograft tumors showed marked decreases in the levels of LC3-II and p-eIF2α, as well as increased levels of cleaved caspase-3 and PARP in the shTIPRL group treated with 2-DG (Fig. 6g). Immunohistochemical analysis revealed a significant decrease in p-eIF2α levels and more TUNEL-positive cells in the shTIPRL group treated with 2-DG (Fig. 6h, i). These findings further indicate that TIPRL knockdown reduced eIF2α phosphorylation, thereby preventing autophagy induction and promoting apoptosis signaling in xenograft tumors. Overall, our results provide compelling evidence of the importance of TIPRL in regulating stress-induced autophagy and indicate that TIPRL is a potentially effective target for lung cancer therapy.

## Discussion

Autophagy is widely studied as a potential therapeutic target in various human diseases^30,31^. Autophagy has been shown to exert pleiotropy, exhibiting anti-carcinogenic, pro-survival, and pro-apoptotic effects in different stages of lung cancers^32^. Recent efforts to develop therapeutic strategies for lung cancer have focused on understanding the role of autophagy as either a tumor-suppressing^33^ or tumor-promoting^34^ mechanism.

Here, we investigated the role of TIPRL protein in lung cancer progression and explored the underlying mechanism whereby TIPRL regulates cancer cell survival/progression. TIPRL knockdown significantly lowered LC3-II accumulation upon various metabolic and ER stress treatments, reduced eIF2α-phosphorylation, and decreased the eIF2α-ATF4 pathway-directed genes. Our results show that TIPRL played a major role in autophagosome formation via the eIF2α-ATF4 pathway and regulated autophagy in stressful environment.

Importantly, we showed that TIPRL knockdown upon metabolic or ER stress reduced mitophagy and aggrephagy activity, eventually leading to cytochrome C release from mitochondria and increased levels of apoptosis through excessive ER stress. TIPRL knockdown reduced ATP recycling and increased cell cycle arrest in EBSS-treated cells, demonstrating that autophagy inhibition severely impaired de novo energy metabolism. This data identifies that the increased apoptotic incidents in TIPRL-knockdown cells were due to autophagy inhibition, subsequent reduction of ATP recycling, and the failure of stress adaptation. Thus, TIPRL-regulated autophagy conferred a survival advantage to cancer cells by allowing them to respond quickly to hostile microenvironmental stresses.

Previously, it was reported that the C-terminal end of TIPRL contains protein-binding sites for proteins such as PP2A and MKK7^13,35,36^. Interestingly, although TIPRL is reported as a PP2A inhibitory protein and interacts with PP2A, neither eIF2α phosphorylation nor autophagy induction are interrupted in PP2A depleted lung cancer cells. Furthermore, even in the absence of PP2A, siTIPRL was able to decrease the eIF2α phosphorylation and autophagy induction. PP2A phosphatase activity also remains unchanged in TIPRL depleted cells treated with EBSS, whereas okadaic acid (OA) successfully inhibited PP2A phosphatase activity in all experimental groups (Supplementary Fig. S4b-d). These novel findings indicate that TIPRL acts as a major regulator of eIF2α phosphorylation, and PP2A is not involved in the effects of TIPRL. For the first time, we report that the region in TIPRL between residues 168–230 specifically bound to the eIF2α, thereby inducing eIF2α phosphorylation. Unexpectedly, a synthetic peptide mimic of TIPRL bound to eIF2α and acted as a competitive inhibitor of the full-length TIPRL protein. These results indicate that the TIPRL-mimic region has a potential to inhibit eIF2α phosphorylation and suppress autophagy. Overall, our results demonstrate that autophagy activated by the eIF2α-ATF4 pathway is selectively regulated by TIPRL.

Cancer cells are often subjected to starvation and hypoxic stresses and have high metabolic demands. Through hanging-drop culture, our results reveal that TIPRL knockdown inhibited spheroid formation of tumor cells exposed to microenvironmental stresses. In addition, TIPRL overexpression and high levels of eIF2α phosphorylation were positively correlated with the malignancy of the cancer. Therefore, it is highly likely that cancer cells with elevated TIPRL expression levels exhibit a greater tendency to form malignant tumors. Accordingly, we found that TIPRL knockdown enhanced the growth-inhibitory effects of 2-DG in xenograft tumors in a mouse model. As shown in Fig. 6f, in the shCon group, treatment with 2-DG showed increased autophagy activity and did not significantly inhibit tumor growth (0.554 g to 0.612 g; +10.5%), indicating stress-induced autophagy helped cancer cell survival upon 2-DG treatment. These results are supported by a previous study, which reported that 2-DG reduced the antitumor efficacy of erlotinib against head and neck squamous cell carcinoma by activating ER stress-induced autophagy^37^. When compared with control mice, shTIPRL group mice showed tumor growth inhibition (0.554 g to 0.33 g; −40.4%), while 2-DG treatment in shTIPRL group mice further increased the degree of inhibition (0.554 g to 0.204 g; −63.2%). These observations indicate that shTIPRL and 2-DG treatment had a synergistic effect on the growth-inhibitory effects of 2-DG and caused a higher level of apoptosis in cancer cells. Moreover, our results showed that treatment of TIPRL-depleted cells either with chloroquine or tunicamycin resulted in the apoptosis by autophagy inhibition. Overall, our data presents compelling evidence that TIPRL overexpression is closely related to tumor malignancy and that anticancer drugs accompanied by TIPRL ablation could be an effective approach to treatment.

**Fig. 7:**
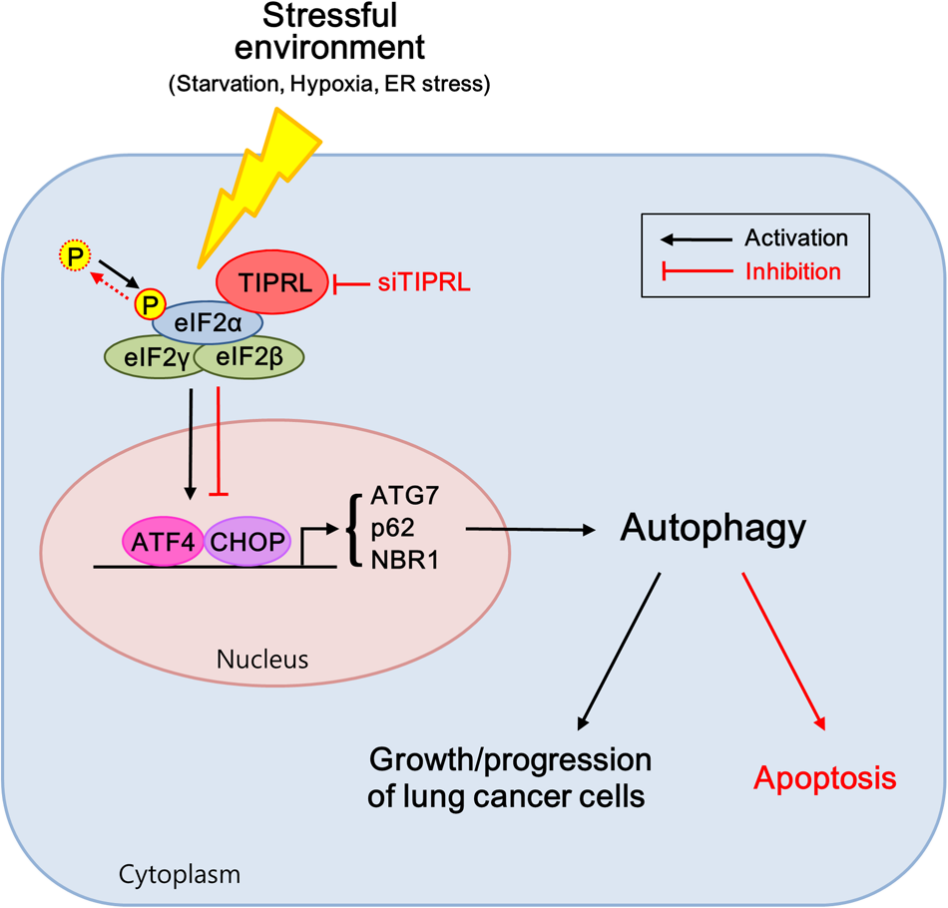
Proposed mechanism of TIPRL-induced autophagy in lung cancer under stressful environment. When TIPRL overexpressing lung cancer cells were exposed to metabolic stress, TIPRL binds to and phosphorylates eIF2α, inducing autophagy by upregulating stress-induced transcription factors, such as CHOP and ATF4, and then accelerating growth and progression of lung cancer cells. Conversely, when TIPRL is depleted, the stressed cells undergo apoptosis because of non-interaction between TIPRL and eIF2α, which in turn downregulates stress-induced transcription factors, eventually inhibiting autophagy.

Overall, the results of this study show that TIPRL binds with and phosphorylates eIF2α, activating the eIF2α-ATF4 pathway. This increases the ability of cancer cells to resist metabolic stresses and may contribute to the development of malignancy through autophagy machinery. In contrast, TIPRL ablation resulted in a substantial decrease of autophagy induction, which led to the reduction of cancer cell survival and enhanced cell death (Fig. 7). Our findings suggest that induction of metabolic and ER stresses coupled with the inhibition of autophagy by TIPRL ablation could be a highly selective and novel therapeutic approach for treatment of various types of cancer.

## Material and methods

### Preparation of patients’ tissue samples

One hundred seventy nine of Paired lung tissue samples including lung cancer and nontumor tissue were collected at Chungnam National University of Medicine (Daejeon, South Korea), to have enough sample size with statistical significance. All patients consented about providing samples before the surgery. The collected tissues were classified according to Edmondson and Steiner. Statistical correlation data were analyzed with SPSS software (Version 13.0; SPSS Inc., IL, USA). Group comparisons of categorical variables were done by the linear by linear association. Lung Tumor and Normal Tissue Array was purchased from BioChain (CA, USA; T8235732-5l). Human subjects study protocol was approved by Ethics Committee of Chungnam National University.

### Cell culture

A549 and H1299 cell lines were maintained in RPMI 1640 and the 293T cell line was maintained in DMEM, supplemented with 10% fetal bovine serum, 1% Penicillin/Streptomycin in a 37 °C humidified incubator with 5% CO_2_. For hypoxia experiments, cells were incubated in 1% O_2_ for indicated hours.

### Chemicals and materials

Tunicamycin (T7765), Chloroquine diphosphate (C6628), 2-deoxy-D-glucose (D6134), and okadaic acid (O9381) were purchased from Sigma Aldrich (MO, USA). Salubrinal (324895) was purchased from Calbiochem (CA, USA). EBSS (14155-063) was purchased from Gibco (MD, USA). The pathscan phospho-eIF2α Sandwich ELISA Kit (Cell signaling technology, MA, USA; 7286C) was used for quantification of phosphorylation of eIF2α, and ATP Assay Kit (abcam, MA, USA; AB83355) was used to detect cellular ATP levels.

### siRNA transfection

Small interfering RNA containing TIPRL silencing sequence and control sequence were purchased from STpharm (Seoul, Republic of Korea) and ATG7 silencing sequence was purchased from Santa Cruz (CA, USA). 50 nM of each siRNA were transfected using Lipofectamine RNAiMAX reagent (Invitrogen, CA, USA; 13778150), following manufacturer’s instruction. siRNA sequences are following; siCon sense: 5′-AUG AAC GUG AAU UGC UCA ATT-3′, antisense: 5′-UUG AGC AAU UCA CGU UCA UTT-3′ and siTIPRL sense: 5′-CCU AAU GAA AUA UCC CAG UAU UU-3′, antisense: 5′-AUA CUG GGA UAU UUC AUU AGG UU-3′.

### Plasmid, truncated mutant’s transfection

GFP-LC3 plasmid was purchased from Addgene (MA, USA). The full-length cDNA of human TIPRL, eIF2α, eIF2β, and eIF2γ were purchased from the KOREA HUMAN GENE BANK (Daejeon, Republic of Korea). TIPRL was subcloned into the HA tagged pCGN vector and eIF2α, eIF2β, and eIF2γ were subcloned to GST tagged pEBG vector to construct the HA-tagged and GST-tagged plasmids, respectively. Truncated mutations of TIPRL containing indicated amino acid length were constructed and subcloned into HA tagged pCGN vector. For GST pull down and immunoprecipitation, plasmids were transfected into cells using Lipofectamine 2000 (Invitrogen, CA, USA; 1668019) following manufacturer’s protocol. In rescue experiments of TIPRL, cells were transfected with siCon or siTIPRL and then the media was changed after 6 h of transfection. After 24 h, HA-tagged TIPRL plasmid were transfected into cell using FUGENE 6 (Promega, WI, USA; E2691) following the manufacturer’s description.

### Antibody and western blotting

The anti-LC3B antibody (L7543) was purchased from Sigma-Aldrich (MO, USA); HA (LF-MA0048) and GAPDH (LF-PA0212) antibodies were purchased from Ab frontier (Seoul, Republic of Korea); phospho-GCN2(T899) (ab75836), HSP60 (ab46798), Cytochrome c (ab76107), FITC (ab19224) antibodies were purchased from abcam (MA, USA); P62 (610832), HIF1α (610959) antibodies were purchased from BD bioscience (CA, USA); TIPRL antibody (a300-663a) was purchased from bethyl (TX, USA); Bip (3183), phosphor-mTOR(s2448) (5536), mTOR (2983), p-p70 s6k(T389) (9234), p-4e-bp1(T37/46) (2855), p-eIF2α(S51) (9721), eIF2α (9722), PERK (3192), MKK7 (4172), Caspase-8 (9746), Caspase-3 (9665), PARP (9542) antibodies were purchased from Cell Signaling Technology (MA, USA); β-actin (sc-47778), ATG7 (sc-33211), GCN2 (sc374609), ATF4 (sc-200), phospho-PERK(T981) (sc-32577), GST (sc-138), PP1 (sc-7482), GADD34 (sc-8327), normal mouse IgG (sc-2025), normal rabbit IgG (sc-2027), TOM20 (sc-17764), Nbr1 (sc-130380), PP2A-Cα/β (sc-6110), Goat-anti Rabbit IgG-HRP (sc-2004), Goat-anti Mouse IgG-HRP (sc-2031), Donkey-anti Goat IgG-HRP (sc-2033) antibodies were purchased from Santa Cruz (CA, USA).

For western blotting, cells were harvested and lysed with RIPA buffer (150 mM NaCl, 20 mM Tris-HCl pH 7.4, 2 mM NaF, 2 mM EDTA, 5 mM Sodium orthovanadate, 1% Triton X-100, 1 mM PMSF, protease inhibitior cocktail) and incubated on ice for 10 min then centrifuged at 13,000 rpm for 15 min and supernatant was collected. Protein concentration was determined using the BCA assay (Thermo Scientific, MA, USA). After quantification, lysates were mixed with 2× sample buffer with β-mercaptoethanol and boiled in 95 °C for 10 min. Samples were subjected to SDS-PAGE gel and transferred to Nitrocellulose membrane. Membranes were blocked with 5% non-fat skim milk in TBS with 0.1% Tween-20 for 1 h then incubated with primary antibodies (1:1000) in 4 °C overnight. Then membranes were washed with TBST for 10 min for 3 times and incubated in secondary antibody in TBST (1:3000) for 1 h at room temperature. After washing, ECL solution was added to membrane and chemical luminescence was detected using LAS-4000 (GE, WI, USA).

### Primer and PCR

Total RNA from cancer cells was extracted using RNAeasy Mini Kit (QIAGEN, CA, USA) following manufacture’s instruction. One microgram of extracted RNA were reverse transcribed into cDNA using AccuPower RT PreMix (Bioneer, Daejeon, Republic of Korea; K-2041-B) and then used for semi-quantitative PCR using AccuPower HotStart PCR PreMix (Bioneer; K-5051) following manufacturer’s protocols. All of the used primer sequences are described in the Supplementary Table. S2. The PCR-amplified products were subjected to electrophoresis through 1% agarose gel.

### Proliferation

Cells were seeded in a 96 well plate at the density of 5 × 10^3^ cells and incubated for 3 days. We prepared 4 individual wells/group for the calculation of the standard deviation. For MTT assay, 2 mg/mL of 3-(4,5-dimethylthiazol-2-yl)−2,5-diphenyltetrazolium was dissolved in PBS. Each day cells were incubated for 2 h with 10 µl of MTT in 100 µl of each well. After removal of media, insoluble purple formazan was dissolved with DMSO and absorbance was measured at 562 nm.

### Go and pathway analysis

David and STRING were used for analysis of Go (http://www.geneontology.org) and functional annotation clustering analysis and Kyoto Encyclopedia of Genes and Genomes (KEGG) (http://www.genome.jp/kegg). Using Protein Analysis through Evolutionary Relationship (PANTHER) (http://www.pantherdb.org), regulated genes associated with molecular process, biological functions and protein classes were mapped.

### TEM

The cells were fixed in 2.5% paraformaldehyde-glutaraldehyde buffer with 0.1 M phosphate (pH 7.2) for 2 h. After fixation in 1% osmium tetroxide for 1 h and dehydration in graded ethanol and propylene oxide, samples were embedded in Epon-812. Sections made by ultramicrotome using ULTRACUT E (Leica, Vienna, Austria) were stained with lead citrate and uranyl acetate then examined using electron microscope CM20 (Philips, Amsterdam, Netherlands).

### Immunohistochemistry

We used DAKO EnVision System (Dako, CA, USA; 4010) for Immunohistochemistry and following solutions are from DAKO. Tumors dissected from mice were fixed in 10% formalin solution, embedded in paraffin and sectioned. After deparaffinization, antigens were retrieved by heating the sections in Target Retrieval Solution (pH 9.0). To eliminate endogenous peroxidase, sections were blocked with Peroxidase Blocking Solution for 10 min. After 30 min of protein blocking, sections were incubated with anti-TIPRL and anti-phospho-eIF2α antibody (500:1) overnight. After washing with PBS, secondary antibody reaction was carried out using DAKO Labeled Polymer HRP anti-rabbit for 1 h. Reaction was visualized by treatment with 3,3′-diaminobenzidine (DAB) stainer. Optimal incubation time of DAB reaction was observed then sections were immediately washed and dehydrated in gradual concentration of ethanol and finally in xylene. Sections were observed with a microscope after mounting.

### Immunoprecipitation

A549 and H1299 cell lines were lysed with Pierce IP lysis buffer (Thermo scientific, MA, USA; 87787) with Halt Protease and Phosphatase Inhibitor Cocktail (Thermo scientific, MA, UA; 78444). After quantification, lysates were used for immunoprecipitation using anti-HA or anti-TIPRL antibodies and protein G-agarose beads (Roche, IN, USA; 11719416001). After incubation with indicated antibodies and protein G-agarose beads for 2 h at 4 °C, immunoprecipitates were washed with 1× TBS (Tris Buffered Saline). Then, immunoprecipitates were subjected to western blotting using indicated antibodies.

### Glutathione S-transferase pull down

293T cells were cotransfected with expression vectors for p-glutathione S-transferase (GST)-tagged-eIF2α, eIF2β and eIF2γ and HA tagged-pCGN-TIPRL plasmids. GST was precipitated from cell lysates. The precipitates were western blotted with indicated antibodies.

### In vitro kinase assay

Recombinant human eIF2α proteins (0.3 µg; Enzo, NY, USA; ADI-KPR-CP132-0050) were incubated with or without recombinant human GCN2 proteins (0.1 µg; abnova, CA, USA; P5554). And gradual concentration (1 µg and 2 µg) of BSA or recombinant human TIPRL proteins (in house; produced in E.coli (BL21 (DE3)) using bacterial expression vector pET21a (C-His) and purified by affinity chromatography using Ni-NTA resin) were added. TIPRL mimic peptide (174–188 amino acids) and Negative Control peptide (86–100 amino acids) were custom made by Peptron (Deajeon, Republic of Korea). Different concentration of peptides were incubated with recombinant human GCN2, eIF2α and TIPRL proteins. After 10 min of reaction at room-temperature with 10× kinase buffer and 10 mM ATP, reaction mixture was subjected to SDS-PAGE and western blotted using anti-phospho-eIF2α antibody. Loading control was confirmed using Ponceau S staining. We repeated this experiment three times to calculate the standard deviation and measured the relative intensity.

### Immunocytochemistry

Cells seeded in a 4-well chamber slide were washed and fixed in 4% paraformaldehyde for 30 min at room temperature, permeabilized in 0.5% Triton X-100 for 5 min and blocked in 5% bovine serum albumin for 30 min. Fixed and permeabilized cells were incubated with TIPRL and eIF2α antibodies (500:1) overnight at 4 °C. Cells were washed and stained using Alexa Fluor-conjugated secondary antibodies (100:1) (Life Technologies, CA, USA; A11008). Nuclei were stained with 4, 6′-diamidino-2-phenylindole dihydrochloride (DAPI) (Vector Laboratories, CA, USA; H-1200). Fluorescent images were captured using a fluorescence microscope.

### Mitophagy monitoring assay

A549 cells were transfected with GFP-LC3 transfected into cells following transfection of siCon and siTIPRL. Then Cells were fixed and permeabilized as described before and incubated with anti-Tom20 antibody (500:1) overnight at 4 °C. Colocalization of GFP-LC3 and Tom20 was observed using fluorescence microscope.

### Aggrephagy monitoring assay

Cells were seeded and transfected with siCon or siTIPRL. Then cells were harvested in Triton X-100 lysis buffer and the supernatants (TX-soluble fraction) were collected. The pelleted material was washed with phosphate buffered saline and extracted with SDS lysis buffer (TX-insoluble fraction). Each fraction was analyzed by SDS-PAGE and western blotting

### FACS

FITC Annexin V Apoptosis Detection Kit (BD Biosciences, CA, USA; 556547) was used for quantification of apoptosis. Cells were harvested and washed with cold PBS, re-suspended with 200 μl of binding buffer [10 mM 4-(2-hydroxyethyl)-1-piperazineethanesulfonic acid (HEPES)-NaOH pH 7.4, 140 mM NaCl, and 2.5 mM CaCl2]. Then, samples were incubated with 5 μl of annexin V conjugated with FITC for 10 min at room temperature in the dark. Cells were washed with binding buffer, stained with propidium iodide (PI) and analyzed using flow cytometer and analysis software (FacsCalibur; BD bioscience, CA, USA).

### Cell cycle analysis

Cells were harvested and washed with cold PBS, then fixed in 70% Ethanol for overnight in −20 °C. RNase A (10 mg/ml) was treated for 1 h at 37 °C and pellets were re-suspended in propidium iodide (PI; 50 μg/ml) solution. Next, samples were analyzed using flow cytometer (FacsCalibur; BD bioscience, CA, USA).

### Spheroid culture

A549 and H1299 cell lines were cultured using hanging drop system. siCon or siTIPRL transfected cells were hanged at the cover of 60 mm dish at the concentration of 1 × 10^5^ cells/20 μl with media in dish to humidify the drops. After 24 h of incubation, cells were harvested and subjected to SDS-PAGE or PCR analysis. Spheroid Microplate with Ultra Low Attachment (Corning, NY, USA; 4515) was also used for spheroid culture. Cells were seeded in ULA plate at the concentration of 2 × 10^4^ cells/ well and spheroids were observed using microscope after 3 days of incubation. 24 wells/ group were measured to have enough sample size and to calculate the standard deviation.

### In vivo xenograft assay

Five-weeks-old female BALB/c-nude mice were purchased from orient bio (Seoul, Republic of Korea) and maintained in accordance with the guidelines of the Institutional Review Committee for Animal Care and Use, KRIBB. 5 mice/group were subjected to the experiment to have statistical importance. Lentivirus mediated TIPRL shRNA and lentivirus mediated scrambled shRNA were custom made by Korea Institute of Science and Technology (Seoul, Republic of Korea) using siRNA sequences used in this paper then tagged with mCherry. A549 cells (5 × 10^6^ cells in 100 μl PBS) were injected subcutaneously into the left flank of the nude mice. At two weeks post inoculation, when tumors reached approximately 50 mm^3^ in volume, the mice were randomly and blindly divided into two groups and were injected intraperitoneally with or without 1000 mg/kg 2-DG (100 μl in PBS/mouse). At 24 h after injection, 2-DG treated, or untreated group were randomly and blindly divided in half again, injected intratumorally with the lentivirus scramble shRNA or lentivirus mediated TIPRL shRNA (1.5 × 10^8^ PFU/mouse). These procedures were repeated every 3 days until the completion of experiments (for 37 days). Tumor growth was monitored every 3 days, measured in two diameters and tumor volumes were calculated as follows: V = (length of width)^2^ × (length of length)/2. At 37 days after injection, mice were sacrificed, and tumors were fixed in 10% formalin solution for immunohistochemistry analysis.

### PP2A phosphatase activity assay

PP2A phosphatase activity was determined using Serine/Threonine Phosphatase Assay System by Promega (WI, USA) following the manufacturer’s instructions. Cells were lysed and subjected to Sepadex G-25 columns to eliminate cellular free phosphate, then protein concentrations were quantified. PP2A phosphatase activity was determined by detecting free phosphate generated from Ser/Thr Phosphopeptide (RRA(pT)VA). Reaction mixtures including 5ug of proteins, PP2A 5× reaction buffer (250 nM imidazole (pH 7.2), 1 mM EGTA, 0.1% β-mercaptoethanol, and 0.5 mg/ml BSA), and phosphopetide were incubated in 37 °C for 30 min, and acidic molybdate dye was added to stop the reaction. 20 nM of okadaic acid or DMSO were added to reaction mixtures as a control. The absorbance of a molybdate/malachite green/phosphate complex generated from the reaction was measured at 630 nm. We prepared 3 individual wells/group to calculate the standard deviation.

## Supplementary information

Supplementary Figure and Table Legends

Supplementary Fig. S1

Supplementary Fig. S2

Supplementary Fig. S3

Supplementary Fig. S4

Supplementary Fig. S5

Supplementary Table. S1

Supplementary Table. S2

Reproducibility Checklist

Author Contribution
