# Supplementary material for: TIPRL potentiates survival of lung cancer by inducing autophagy through the eIF2α-ATF4 pathway
Source: Cell Death Dis. 2019 Dec 20;10(12):959. doi: 10.1038/s41419-019-2190-0 (PMC6925247; doi:10.1038/s41419-019-2190-0)
Supplement: Supplementary file 1 — Supplementary Figure and Table Legends [file 41419_2019_2190_MOESM1_ESM.docx]

**SUPPLEMENTARY FIGURE LEGENDS**

**Supplementary Fig. S1. TIPRL knockdown changes molecular pathway related to metabolic process.** GO database and PANTHER classification (A), and KEGG pathway (B) were used to analyze the RNAs of A549 cells transfected with siCon or siTIPRL.

**Supplementary Fig. S2. TIPRL depletion inhibits aggrephagy upon metabolic stress.** (A) Detection of aggrephagy using two different lysis buffers (Triton X-100 or SDS lysis buffer) in TIPRL-knockdown A549 and H1299 cells treated with 50 µM chloroquine (left panel) or incubated in EBSS for 2 h (right panel).

**Supplementary Fig. S3. TIPRL depletion inhibits the expression of autophagy related-genes upon metabolic stress.** (A-C) Detection of autophagy related genes using western blots (upper panel) and RT-PCR (lower panel). A549 cells were treated with EBSS time dependently (A). TIPRL-knockdown H1299 cells were incubated with 1 % O2 environment for 6 h (B). TIPRL-knockdown A549 and H1299 cells were treated with Tunicamycin (0.2 µg/ml) for 24 h or Salubrinal (10 µM) for 12 h (C).

**Supplementary Fig. S4. Phosphorylation of eIF2α by TIPRL is independent of PP2A.** (A) In vitro kinase assay of phospho-eIF2α using recombinant eIF2α and GCN proteins with 200 uM of TIPRL-mimic peptides. Phosphorylation of eIF2α and transfection of peptides were detected by Western blots, and recombinant eIF2α protein was stained using Ponceau S. (B) IP assay of cell lysates of A549 cells with anti-TIPRL antibody. (C) Detection of autophagy activity and phosphorylation of eIF2α in A549 and H1299 cells treated with EBSS after transfection with siCon, siTIPRL, and/or siPP2A. (D) PP2A phosphatase activity in A549 and H1299 cells treated with EBSS after transfection with siCon or siTIPRL. 20 nM of okadaic acid (OA) was treated as a specificity control in all experimental groups.

**Supplementary Fig. S5. The viability of A549 cells treated with 2-DG and the weight change of mice.**  (A) Cell viability assay of A549 cells treated with the indicated concentrations of 2-DG after transfection with siCon or siTIPRL. (B) Weights of mice were measured on the first and last days of the experiment.

**Supplementary Table. S1.** The correlation between TIPRL expression and stage in NSCLC patients. P value was calculated from linear by linear associations (n=179).

**Supplementary Table. S2.** Primers used in this study.
