# Supplementary figures and images for: TIPRL potentiates survival of lung cancer by inducing autophagy through the eIF2α-ATF4 pathway
Source: Cell Death Dis. 2019 Dec 20;10(12):959. doi: 10.1038/s41419-019-2190-0 (PMC6925247; doi:10.1038/s41419-019-2190-0)

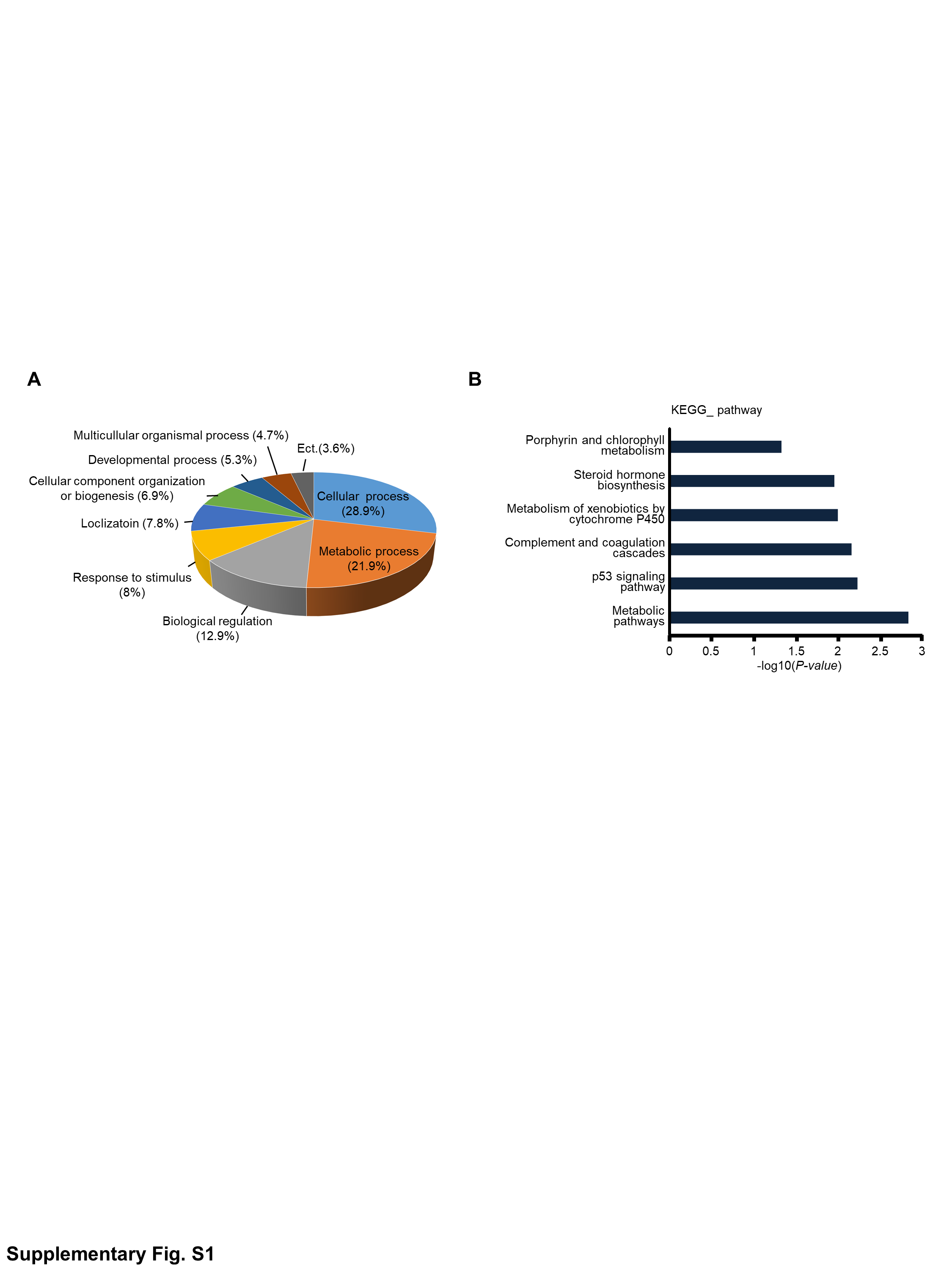

Supplement: Supplementary file 2 — Supplementary Fig. S1 [file 41419_2019_2190_MOESM2_ESM.tif]

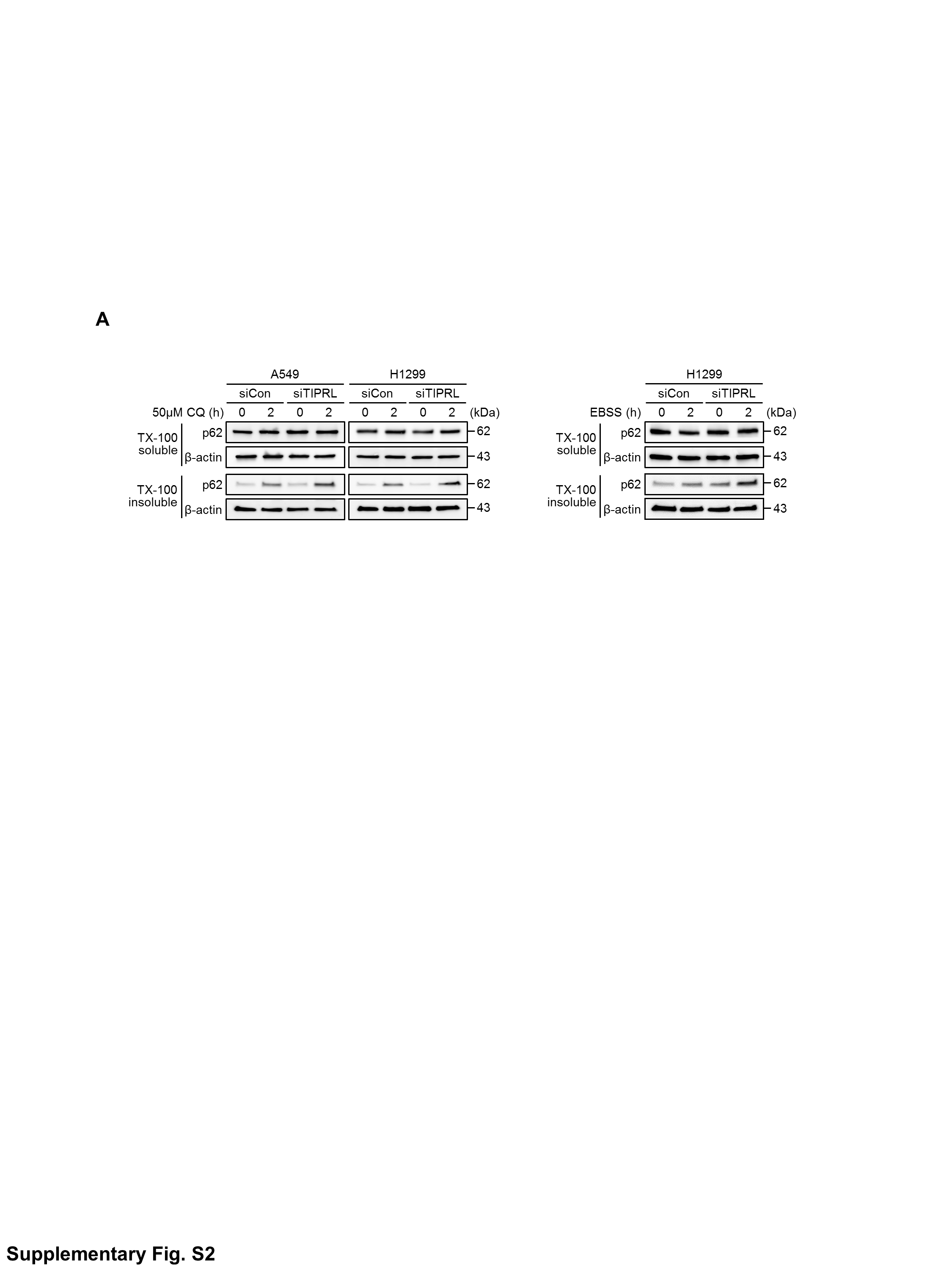

Supplement: Supplementary file 3 — Supplementary Fig. S2 [file 41419_2019_2190_MOESM3_ESM.tif]

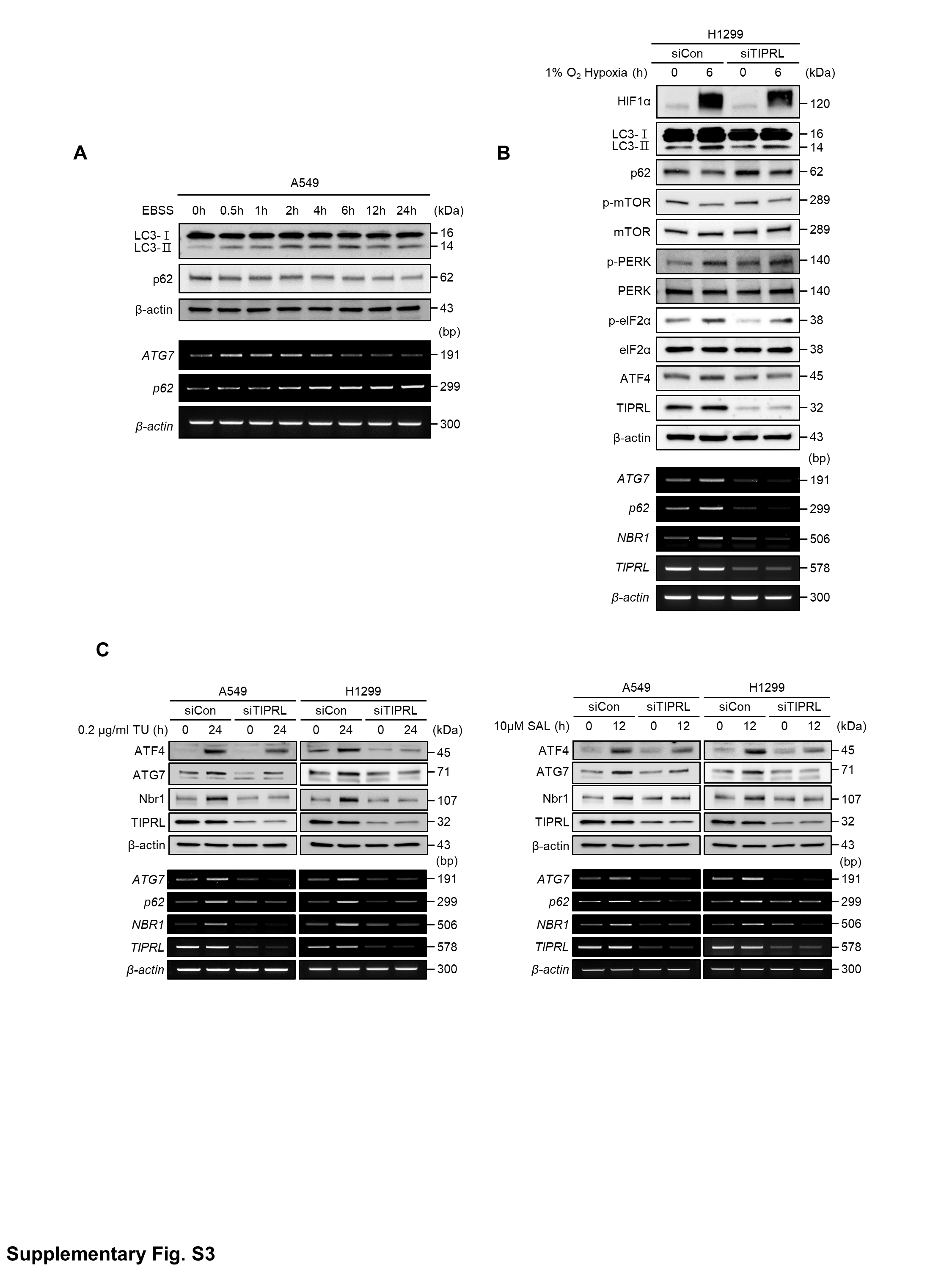

Supplement: Supplementary file 4 — Supplementary Fig. S3 [file 41419_2019_2190_MOESM4_ESM.tif]

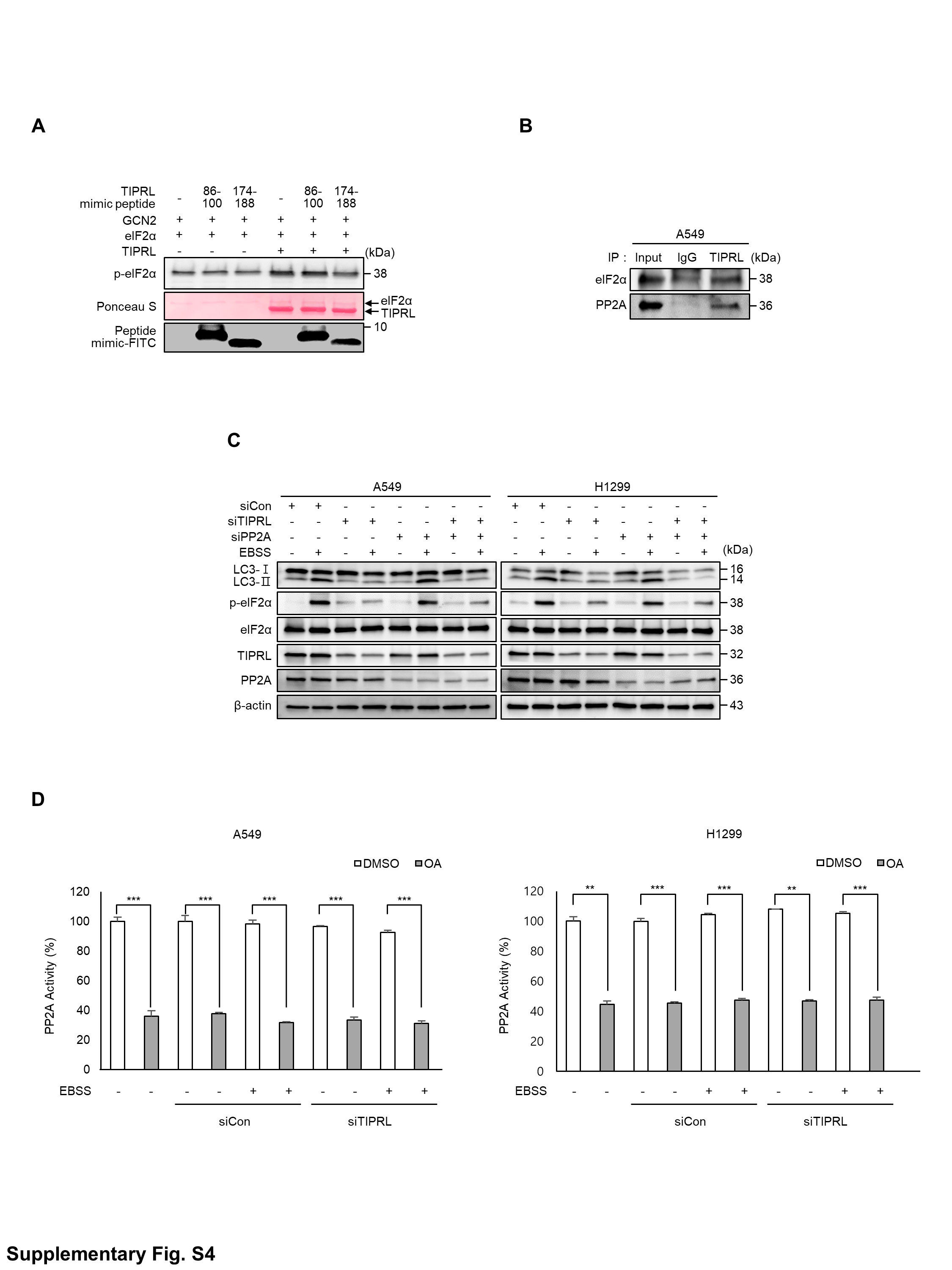

Supplement: Supplementary file 5 — Supplementary Fig. S4 [file 41419_2019_2190_MOESM5_ESM.tif]

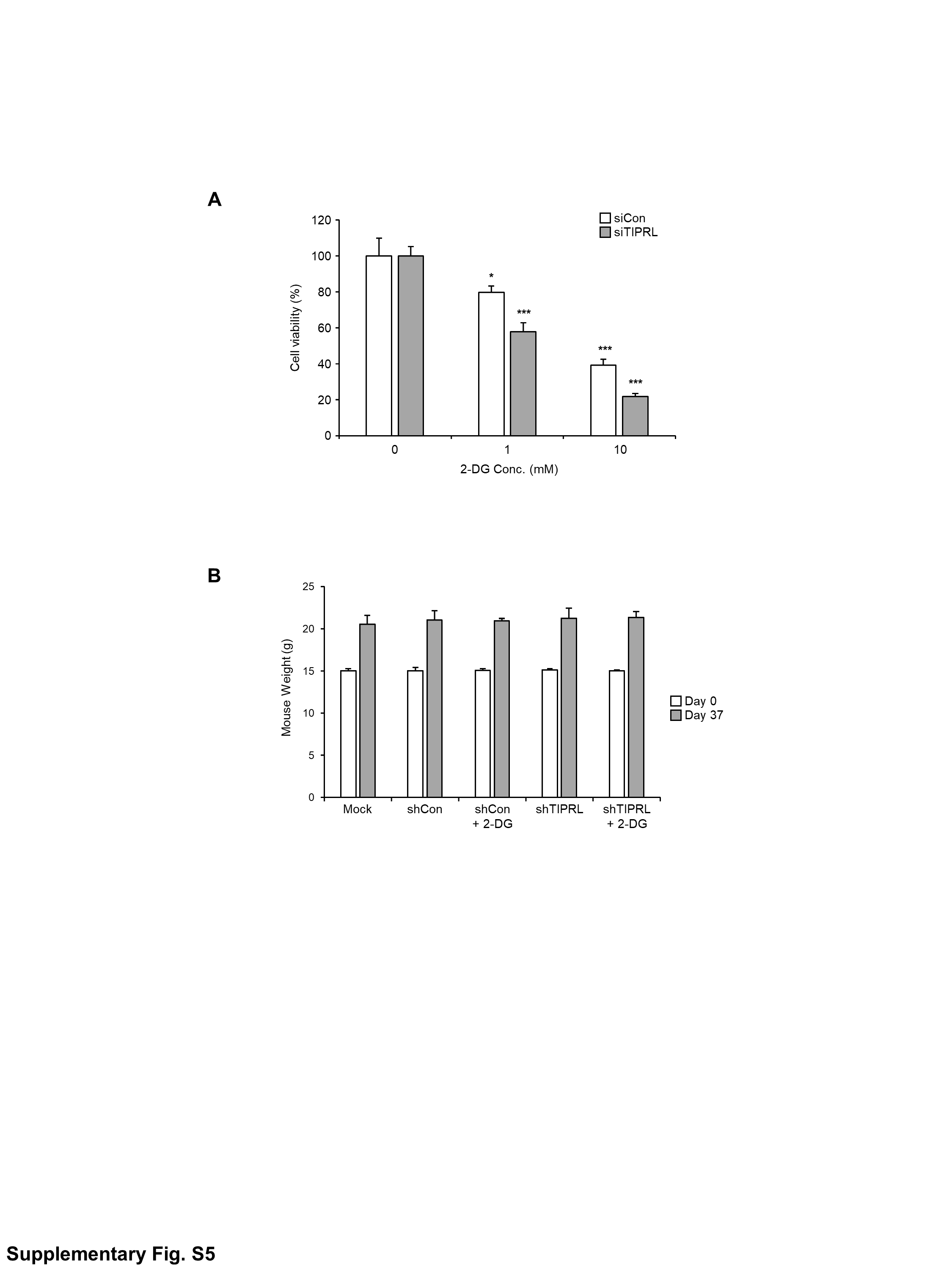

Supplement: Supplementary file 6 — Supplementary Fig. S5 [file 41419_2019_2190_MOESM6_ESM.tif]
