# Supplementary material for: TIPRL potentiates survival of lung cancer by inducing autophagy through the eIF2α-ATF4 pathway
Source: Cell Death Dis. 2019 Dec 20;10(12):959. doi: 10.1038/s41419-019-2190-0 (PMC6925247; doi:10.1038/s41419-019-2190-0)
Supplement: Supplementary file 10 — Author Contribution [file 41419_2019_2190_MOESM10_ESM.pdf]

DECLARATION OF CONTRIBUTIONS TO ARTICLE

ADMC

Manuscript Number:	Journal Name:	
CDDIS-19-1802RR	*Cell Death & Differentiation*	(the 'Journal')

Proposed Title of the Contribution:

TIPRL potentiates survival of lung cancer by inducing autophagy through the eIF2α-ATF4 pathway (the 'Contribution)

Author(s):

Su-Jin Jeon, Jun-Ho Ahn, Debasish Halder, Hyun-Soo Cho, Jung-Hwa Lim, Soo Young Jun, Jeong-Ju Lee, Ji-Yong Yoon, Min-Hyuk Choi, Cho-Rok Jung, Jin-Man Kim, and Nam-Soon Kim (the 'Authors')

For all *CDD* articles, each person named as an author in the published version must be able to show he or she has contributed substantially to the article.

Authorship credit should be based on 1) substantial contributions to conception and design, acquisition of data, or analysis and interpretation of data; 2) drafting the article or revising it critically for important intellectual content; and 3) final approval of the version to be published. Authors should meet conditions 1, 2 and 3.

Any person who cannot be shown to have made a substantial contribution to the article cannot be listed as an author in the final version. The name of any person who is deemed to have made a minor contribution can, however, appear in the Acknowledgments section of the article.

Please complete the table below to indicate the contributions of all named authors to the manuscript.

Author Full Name:	Specification of Contribution to the Manuscript:
Su-Jin Jeon	designed and conducted experiments, analyzed results and wrote the manuscript
Jun-Ho Ahn	designed and conducted experiments, analyzed results and wrote the manuscript
Debasish Halder	advised on data analysis and writing of the manuscript
Hyun-Soo Cho	designed and conducted experiments and analyzed results
Jung-Hwa Lim	designed and conducted animal experiments and analyzed results
Soo Young Jun	advised on data analysis and writing of the manuscript
Jeong-Ju Lee	provided vectors and advised on data analysis
Ji-Yong Yoon	provided vectors and advised on data analysis
Min-Hyuk Choi	designed and conducted the RNA sequencing data and advised on data analysis
Cho-Rok Jung	designed and conducted animal experiments and analyzed results
Jin-Man Kim	designed and conducted clinical experiments and analyzed results
Nam-Soon Kim	designed experiments, analyzed results, wrote the manuscript and supervised all the procedures of experiments

Please complete the table below to indicate the contributions of all named authors to the figures.

Figure 1:

A: JMK generated the W.B data and assembled the panel.
B: JMK generated the immuno-histochemistry data.
C: SJJ and JHA generated the MTT data, and SJJ generated the W.B data.
D: SJJ and JHA generated the W.B data.
E: SJJ and JHA generated the FACS data.
SJJ and JHA assembled every panel in Figure 1.

Figure 2:

A, B, C: SJJ and JHA generated the W.B data.
D: SJJ and JHA generated and analyzed GFP-LC3 puncta assay data, and made the graph.
E: SJJ and JHA prepared sample for the TEM.
F: SJJ and JHA generated the W.B data.
SJJ and JHA assembled every panel in Figure 2.

Figure 3:

A: SJJ and JHA generated the W.B data.
B: SJJ and JHA generated Immunocytochemistry data.
C: SJJ and JHA generated ATP assay data.
D: SJJ generated the cell cycle experiment.
SJJ and JHA assembled every panel in Figure 3.

Figure 4:

A, B: SJJ and JHA generated the W.B data.
C: SJJ and JHA generated the ELISA assay data.
D: SJJ and JHA generated the W.B data.
E: SJJ and JHA generated the W.B data and the RT-PCR data.
F: SJJ and JHA generated the W.B data.
G: HSC generated the immunohistochemistry data.
SJJ and JHA assembled every panel in Figure 4.

Figure 5:

A: JHA generated the upper panel of IP data, and SJJ generated lower panel of IP data.
B: JHA generated the IP data.
C: HSC generated and analyzed the Immunocytochemistry data, and made the graph.
D: SJJ and JHA generated the IP data, and JJL and JYY provided vectors used in the experiments.
E: JHA generated the IP data and SJJ made the panel of subcloned fragmentes of TIPRL.
F: SJJ and JHA generated the IP data.
G, H: HSC generated and analyzed the in vitro kinase assay data, and made the graph.
SJJ and JHA assembled every panel in Figure 5.

Figure 6:

A, B: SJJ and JHA generated the W.B data and RT-PCR data.
C: SJJ and JHA generated and analyzed the microscopy images data, and made the graph.
D, E, F: JHL and CRJ carried out xenograpft experiments, tracked tumor volume and mass, and made the graph.
G: SJJ and JHA generated the W.B data.
H: SJJ and JHA generated the immunohistochemistry data.
I: SJJ and JHA generated and analyzed the tunel assay data, and made the graph.
SJJ and JHA assembled every panel in Figure 6.

Signed for and on behalf of the Author(s):	Print Name:	Date:
Nam-Soon Kim	Nam-Soon Kim	6th, October, 2019
